# Similar collagen distribution in full-thickness skin grafts in intraperitoneal and onlay positions, an experimental mice-study

**DOI:** 10.1007/s10029-022-02664-0

**Published:** 2022-09-01

**Authors:** A. Winsnes, M.-L. Ivarsson, P. Falk, U. Gunnarsson, K. Strigård

**Affiliations:** 1 Department of Surgical and Perioperative Sciences, Surgery, Umeå University, Daniel Naezéns väg, 901 87 Umeå, Sweden; 2 Department of Surgery, University of Gothenburg, Gothenburg, Sweden

**Keywords:** FTSG, ECM, MMP, Collagen, Parastomal, Hernia

## Abstract

**Purpose:**

Autologous full-thickness skin grafting (FTSG) has the potential to become an option in abdominal wall repair. An understanding of tissue remodelling in the extracellular matrix (ECM) is crucial as this interplay determines such parameters as tissue strength and flexibility. This cross-sectional preclinical laboratory study in mice provides information on the distribution of collagen types and matrix metalloproteinases (MMPs) in the ECM of FTSGs in the intraperitoneal and onlay positions compared with internal controls. The aim was to evaluate morphologic changes after tissue remodelling and repair in FTSGs applied in the two positions and to detect any adverse host response.

**Methods:**

ECM components were evaluated as follows: qualitative examination of collagen bundle thickness using Picrosirius Red staining (collagen types I, III and IV); and evaluation of collagen types IV and V, as well as MMPs 1, 8 and 9 using immunohistochemical staining. Full-thickness grafts transplanted between female twin mice were examined as this best mimics autologous transplantation.

**Results:**

At 8 weeks, FTSGs in the intraperitoneal position did not show any noticeable differences in morphologic appearance to those in the onlay position. Both intraperitoneal and onlay FTSGs showed increases in the amount of thick collagen bundles compared to internal controls. No correlation was seen between distribution of MMPs 1, 8 or 9 and distribution of collagen types I, III, IV or V.

**Conclusion:**

This preclinical study shows that FTSGs in both intraperitoneal and onlay positions are possible application site options and, by extension, promising application site options for abdominal wall reinforcement in hernia surgery. Clinical studies in humans are required to confirm these findings.

## Introduction

Implantation of synthetic mesh in hernia repair is standard practice, but several studies have shown this technique to be associated with severe adverse events such as chronic pain, gut erosion, intestinal obstruction, enterocutaneous fistula formation and infection [1–4]. These complications together with the need for prosthetic material suitable for use in contaminated fields have led to the introduction of biologic graft materials. These are marketed as a better alternative in complex cases but are extremely expensive. Furthermore, clinical performance has been disappointing with high recurrence rates, questionable tolerance to infection, and late immunologic reactions which led the European consensus conference in Berlin to declare them inadvisable as of January 2016 [5]. This, together with patient demand for non-synthetic prosthetic material, calls for a new repair strategy.

With this in mind, our research group performed an RCT (Randomized Controlled Trial) comparing full-thickness skin graft (FTSG) applied on the anterior rectus sheath (onlay) with synthetic mesh in the best possible position with synthetic mesh in the best possible position (sublay, onlay or intraperitoneal depending on patient properties), as reinforcement material in the repair of giant incisional hernia in humans [6]. Autologous tissue has many advantages including making decellularization superfluous as well as tolerating bacteria well [7], and has superior tensile strength compared to synthetic and biologic implant materials [8]. Outcomes in the RCT at 3 months were similar for the main endpoint “surgical site complication”, and at one-year follow-up results were similar regarding adverse events, recurrence, pain, muscle strength, patient satisfaction and esthetic outcome [9]. Biopsies taken several years later during abdominal surgery for reasons other than hernia repair, showed remodulation of the FTSG to normal fascia [6].

Intraperitoneal mesh position is today used in 20–30% of laparoscopic repair of inguinal hernia and in repair of ventral and parastomal hernia [10], and it is important to develop novel contexture-compatible prosthetic materials for use in the intraperitoneal position [11].

This translational study was executed in answer to clinical demands where implantation of synthetic reinforcement material implies a high risk for material related complications or where high recurrence rate is seen, as for parastomal hernias.

Research on intraperitoneal application of FTSG in humans must be based on a translational concept that includes both animal and morphologic studies. Winsnes et al. were the first to systematically evaluate FTSG in the intraperitoneal position in mice compared to the onlay position [11]. Onlay position was used since the abdominal wall muscles in mice are too thin to allow for sublay positioning of the graft. Both positions showed survival of all FTSG and few adhesions at 8 weeks and no FTSG-related complications. Furthermore, higher survival rates were seen in the subgroup of same sex donator/recipient, that is FTSG from female donors to female recipients [11]. Confirmation of cell survival and low inflammatory reaction were verified in a cross-sectional study after sacrifice at 8 weeks postoperatively. In the present study evaluating autologous FTSG grafting, same sex transplantation was considered the most representative, being genetically identical except for one enzyme [11], and avoiding the sex-chromosome as a potential immunological target [12]. This group was thus included for further morphological evaluation.

To increase our understanding of the biologic mechanisms behind hernia recurrence, studies on the strength and endurance of connective tissue are crucial. The biostructure of the collagen framework in the abdominal wall is an important determinant of these properties.

The aim of this preclinical laboratory study was to describe the morphology of tissue remodeling after implantation of FTSG in intraperitoneal and onlay positions in terms of distribution of specific collagen types, and to assess any differences between the two positions. A secondary aim was to investigate extracellular matrix remodeling including the distribution of MMPs in relation to collagen types.

The main hypothesis was that there are no differences in morphologic changes seen in FTSGs in the intraperitoneal and those in the onlay position. A second hypothesis was that there is an increase in thick collagen bundles and degradation of adnexal structures in FTSGs that does not occur in their internal control (laterally juxta-positioned abdominal wall tissue). A third hypothesis was that there is co-localization of MMPs with their respective collagen substrates.

## Materials and methods

Surgical intervention was performed on mus musculus strain C57BL/6 median age 6 months (4–11 months), anesthetized with intra-abdominal injection of Zolazepam, Tiletamine, Xylazine and Fentanyl, as described in Winsnes et al.[11]. Briefly, after razor-removing hair from a 10 × 10 mm area of skin the donor, the FTSG was grafted through aseptic technique to a position either onlay (on the anterior rectus sheath) or intraperitoneal (intraabdominal on the peritoneum) in a genetically identical female twin, in a consecutive setting without randomisation.

During surgery mice were put on a temperature pad, with eye-gel applied, and rehydration together with analgetics (Temgesic) was administered at the end of each procedure. There were four animals in each group, adding the donor mouse a total of nine animals were used for this experiment. The donor was euthanised after the procedure; still under anaesthesia translocated to a carbon dioxide chamber. As the donor tissue was immunologically identical to the recipient, no decellularization process was required. Time between harvest and placement was kept to a minimum, between 10–30 min, during which the FTSG was hydrated in cold isotonic phosphate-buffered saline. FTSG fixation in the abdominal wall was performed with four coated 6-0 or 7-0 vicryl absorbable sutures, one in each corner. It was decided not to establish an artificial abdominal wall hernia as this would have introduced other sources of error. Furthermore, the aim of this study was only to evaluate morphologic changes in FTSGs applied in intraperitoneal and onlay positions and to detect any adverse host response. Exclusion criteria included any illness that made further participation unethical according to the veterinary. The graft was examined every 2–3 days until sacrifice at 8 weeks, when tissue was gathered for histology, as described by Winsnes et al. [11].

Transplantation between genetically identical females was selected for histology studies as this best represents autologous repair. Female mice were used to increase animal welfare since they tolerated cohabitation during the long follow-up period better than males. It was decided not to use statistical analysis, since the outcome data were mostly semi-quantitative and aimed to explore FTSG behaviour in the two locations.

Ethics: All animal studies were approved by the Animal Care Institutional Review Panel in agreement with FOR-1996-01-15-23 (Oslo University, Oslo University Hospital). The animal experiments were performed between 2015–2016 at the Institute of Basic Medical Sciences, Oslo, Norway, in accordance with EU Directive 2010/63/EU for animal experiments and ARRIVE criteria [13].

### Tissue handling and paraffin embedding

The abdominal wall including the implanted FTSG was gently dissected in a 15 × 15 mm en bloc fashion and fixed in 4% formalin buffer. All biopsies were rinsed in phosphate-buffered solution (PBS, Sigma-Aldrich, St Louis, MO, USA), thereafter dehydrated in increasing concentrations of alcohol (70–99.5%) and finally clarified using xylene (Histolab, Askim, Sweden). Formalin-fixed and paraffin-embedded 4–5 µm tissue slices were mounted on adhesion slides (Superfrost plus and Superfrost ultra plus; Histolab, Askim, Sweden), and dried at 37 °C for 3 h.

### Choice of biomarkers

Both collagen type I and collagen type III are widely distributed in skin. Collagen type I provides strength in tissue, while collagen type III gives multidirectional elasticity. Increasing amounts of collagen type III give rise to a higher proportion of thinner collagen bundles [14]. Collagen types I and III were chosen as major markers, stained with Picrosirius Red and evaluated by cross-polarization light microscopy (CRM).

Collagen types I and III are degraded by the collagenases MMP-1 and MMP-8 [15, 16], and were thus chosen for identification of matrix remodulation of collagen types I and III.

Collagen type IV is a degradation by MMP-1 and was thus chosen to study matrix remodulation of collagen type IV [17].

Collagen type V distribution in skin matches collagen type I distribution and is important in the regulation of fibrillogenesis of collagen type I to heterotypic collagen type I fibrils [18].

Gelatinase MMP-9 degrades both collagen type IV [19], and collagen type V [20].

### Collagen staining with picrosirius red

Labeled glasses were deparaffinized, stepwise rehydrated in xylene and alcohol (99.5–70%) and rinsed in tap water. Slides were incubated in equal parts of Weigert’s Hematoxylin (Solution A + B, Histolab, Göteborg, Sweden) for 10 min, rinsed in tap water for 1 min, followed by a short dip in HCL/EtOH 95% and additional rinse in tap water for 3 min. Staining with Picrosirius Red (0.1%) was done for 30 min in a saturated picric acid solution (Histolab, Göteborg, Sweden), followed by rinse in acetic acid/water (5 mL/1000 mL). Thereafter the preparations were dehydrated in alcohol (70–99.5%), clarified with xylene, and finally mounted with Pertex (Histolab, Sweden). Slides were examined in an Eclipse E800 research microscope using the CRM technique as described by Junqueira et al.[21] To visualize and evaluate the presence of collagen structures a manually rotated 180˚-polarizer was positioned beneath the specimen stage and an analyzer placed above the objectives. Cross-polarization occurs when the raster of the polarizer and analyzer are at 90˚ to each other, thereby preventing light passage through the system and creating a dark field.

### Immunohistochemistry

Labeled glasses were deparaffinized, rehydrated stepwise in xylene then alcohol (99.5–70%) and rinsed twice in 5 mM Tris-buffered saline solution (TBS), pH 7.8 (Sigma-Aldrich, St Louis, MO, USA). Heat-induced antigen retrieval (HIAR) was performed by the glass preparations being heated for 15 min at 95 °C in 0.01 mol/L citrate buffer, pH 6.1 or pH 9 as target retrieval solution (pH 6.1: DAKO S1700, pH 9: DAKO S2368; Dako/Agilent, Kista, Sweden). Following rinsing with TBS, the prepared glass slides were mounted on Shandon cover plates (Thermo Fisher Scientific, USA). Non-specific protein binding was initially done using 5% fat-free dry milk in TBS, followed by endogenous peroxidase blockade using ready-to-use components in the Dual Link System-HRP kit (K4010; DakoCytomation/Agilent) or bought separately as Peroxidase-Blocking Solution (DAKO S2023; Dako Denmark A/S, Glostrup, Denmark), EnVision + System-HRP Labeled Polymer Anti-Rabbit (DAKO K4003; Dako North America, CA 93013 USA) and Liquid DAB + Substrate Chromogen System (DAKO K3468; Dako North America, CA 93013 USA). After TBS wash, primary antibodies were added (Table 1). For each stain, one primary antibody was applied to the test tissue as well as to a negative control.

**Table 1:** Specifications of antibodies used in the immunohistochemistry

Epitope	No	Manufacturer, Lot	Dilution (µg/mL)
Collagen type IV, polyclonal rabbit anti-Collagen type IV	ab6586	Abcam, MA, USA Lot#GR3278425-2	1.25–2.5 µg/mL
Collagen type V, polyclonal rabbit anti-Collagen type V	ab7046	Abcam, MA, USA Lot#GR3248507-7	5.2–10.5 µg/mL
MMP1, polyclonal rabbit anti-MMP1	ab137332	Abcam, MA, USA Lot#GR3180071-24	0.6–0.8 µg/mL
MMP8, polyclonal rabbit anti-MMP8	ab53017	Abcam, MA, USA Lot#GR38794-30	1.2–1.7 µg/mL
MMP9, polyclonal rabbit anti-MMP9	NBP1-57,940	Novus, Bio-Techne Ltd, Abdington, UK Lot#A-1	0.1–0.25 µg/mL
Neg ctrl, IgG fractions from healthy non-immunized rabbits	X-0903	Dako, Denmark Lot#00,068,049	Same protein conc as primary antibody

All dilutions were made in 5% fat-free dry milk in TBS

### Primary antibodies

Primary antibodies and negative controls were incubated overnight at 4˚C and rinsed twice using TBS solution. Following incubation with peroxidase-labeled polymer conjugated to goat anti-rabbit immunoglobulin, the sections were incubated using 3,3’-diaminobenzidine (DAB +) as chromogenic substrate (DAKO K3468, Dual Link System-HRP, Dako, CA, USA). After rinsing with TBS, counterstaining was performed using Harris HTX only and mounted using Pertex (Histolab, Sweden) following dehydration using alcohol (70–99.5%) and clarification with xylene. All immunostaining procedures were performed in several serial dilutions using duplicates for most of the dilutions. Similar techniques have been described for human peritoneal tissue [22, 23]. Immunohistochemical evaluation was performed by photographic documentation using the Eclipse E800 microscope (× 10, × 20, × 40 objectives) connected to CoolPix 995 photographic equipment with an MDC-lens using neutral density filters D, ND2-32 (Nikon Instruments, Amsterdam, Netherlands).

### Evaluation

Evaluation was not blinded, however specimen number and intervention were hidden during evaluation. Specimens from abdominal wall adjacent to FTSG served as internal controls. This was made possible by wide excision of the graft. For IHC, qualitative description of antibody distribution and grade of staining was planned in a semi-quantitative manner; negative (−), weak positive (+) and strong positive (++). To evaluate Picrosirius staining, the relative amounts of thin and thick collagen bundles were compared between intraperitoneal and onlay grafts.

## Results

**Fig. 1:**
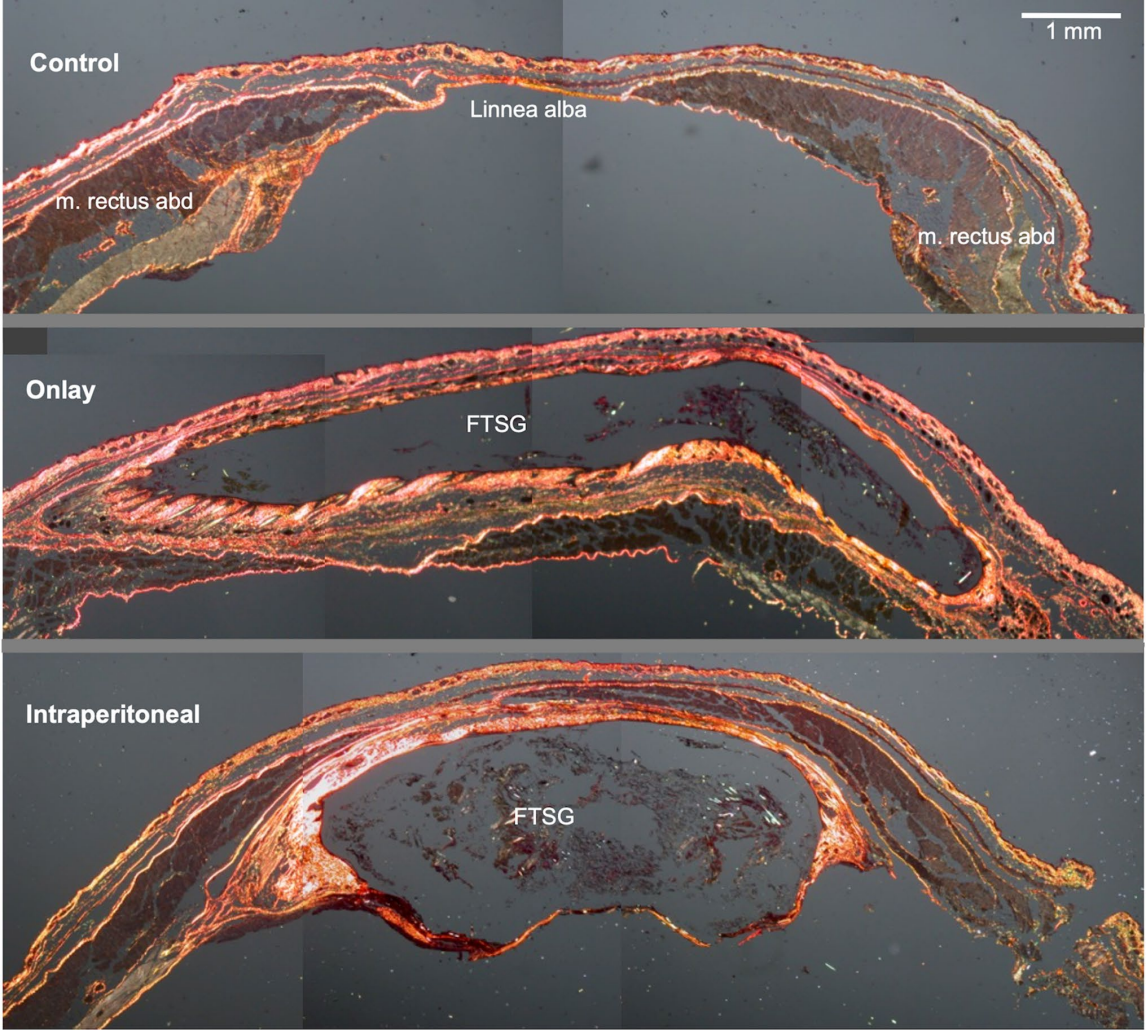
Overview of FTSG in intraperitoneal and onlay position with similar appearance. Picrosirius stain in polarized light, stained slides perpendicular to abdominal wall. Note the thickened dermis and the partial disappearance of skin appendages. From top: Control, onlay, intraperitoneal. *FTSG* Full-thickness skin graft. The size bar equals 1 mm

Representative slides and internal controls from 8 female mice donors and recipients were evaluated. There were no exclusions. FTSG applied in the intraperitoneal as well as onlay position showed similar histologic appearance with no systematic differences. Picrosirius stain levels were similar, as well as one central dermoid cyst with uncertain ground substance and hair follicles present in varying degrees of degradation (Fig. 1).

**Fig. 2:**
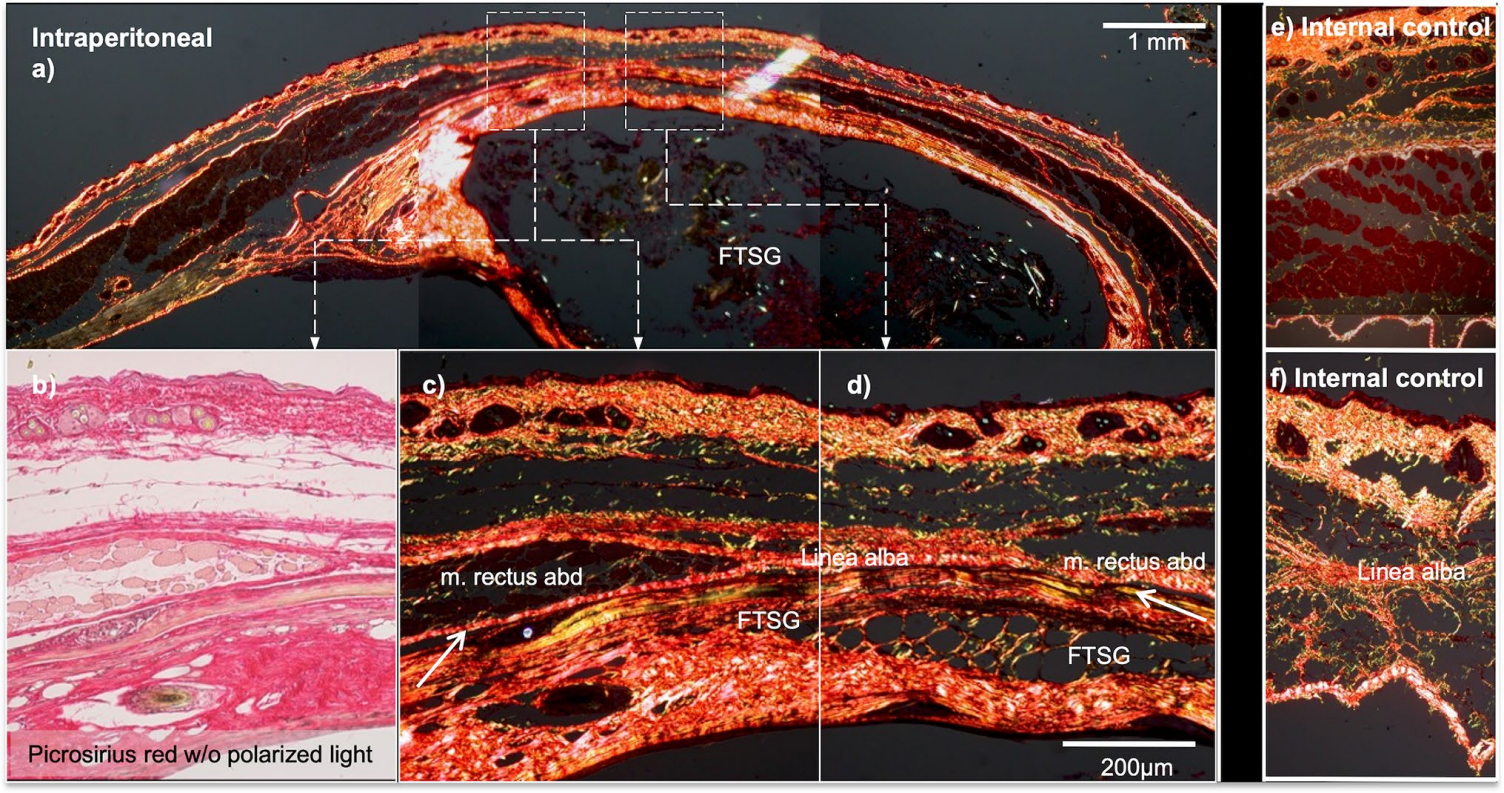
Detail of intraperitoneal FTSG in linea alba area (**d**) and lateral (**b**, **c**) compared to internal controls (**e**, **f**). Transition between FTSG and recipient (arrows). Note m panniculus carnosus muscle in FTSG (**b**,**c**). Picrosirius staining viewed without (**b**) and with (**a**, **c**–**f**) polarized light shows thickened collagen bundles in the FTSG area compared to internal controls (**e**, **f**). The size bar equals 1 mm (**a**) and 200 µm (**b**–**f**)

**Fig. 3:**
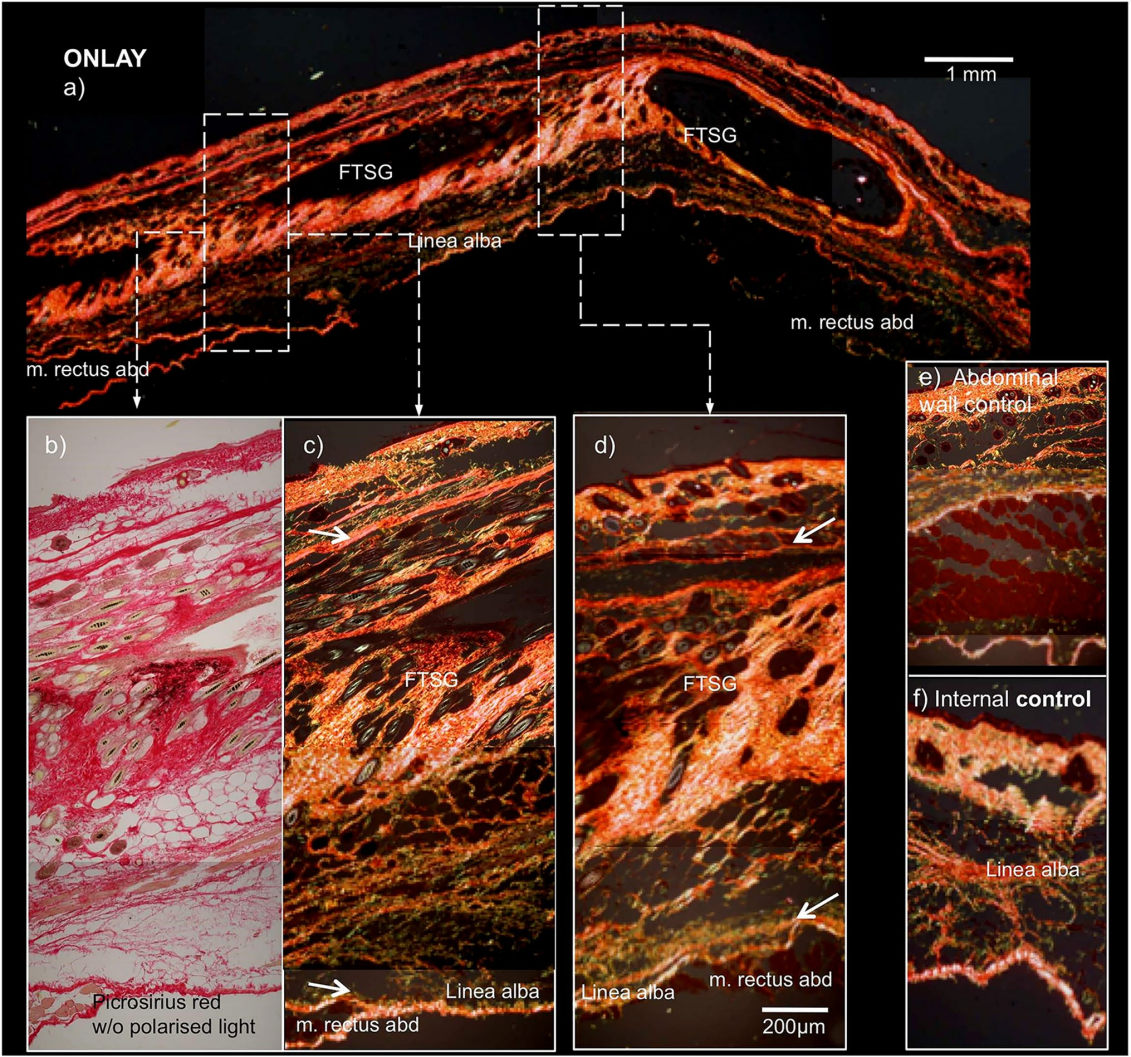
Detail of onlay FTSG in linea alba area (**b**, **c**) and lateral (**d**), compared to internal controls (**e**, **f**). Transition between FTSG and recipient indicated by arrow. Picrosirius staining viewed without (**b**) and with (**a**, **c**–**f**) polarized light shows thickened collagen bundles in the FTSG area compared to internal controls (**e**, **f**). The size bar equals 1 mm (**a**) and 200 µm (**b**–**f**)

Furthermore, grafts from both positions showed similar organization of collagen fibers, with transformation of epidermis and dermis into collagenous/fibrous tissue. Some original tissue architecture remained distinguishable as dermis, musculus panniculus carnosus, connective tissue, epidermis and hair follicles (Figs. 2 and 3).

Compared to the internal controls, FTSG in both intraperitoneal and onlay positions had a slightly thicker dermis and connective tissue with somewhat slimmer musculus panniculus carnosus (Figs. 2 and 3). Low levels of loose connective tissue fibers (green Picrosirius) corresponding to collagen III were seen in both intervention groups, compared to internal controls (Figs. 2 and 3). These differences in color remained throughout the cycle while the polarizing filter was turned 360°. Even if the intensities of the colored areas varied to some extent, there was no observed change from green to orange/red.

The extracellular matrix (ECM) around each graft, as visualized by Picrosirius staining, kept its normal morphologic appearance, resembling the internal control (Fig. 3).

**Fig. 4:**
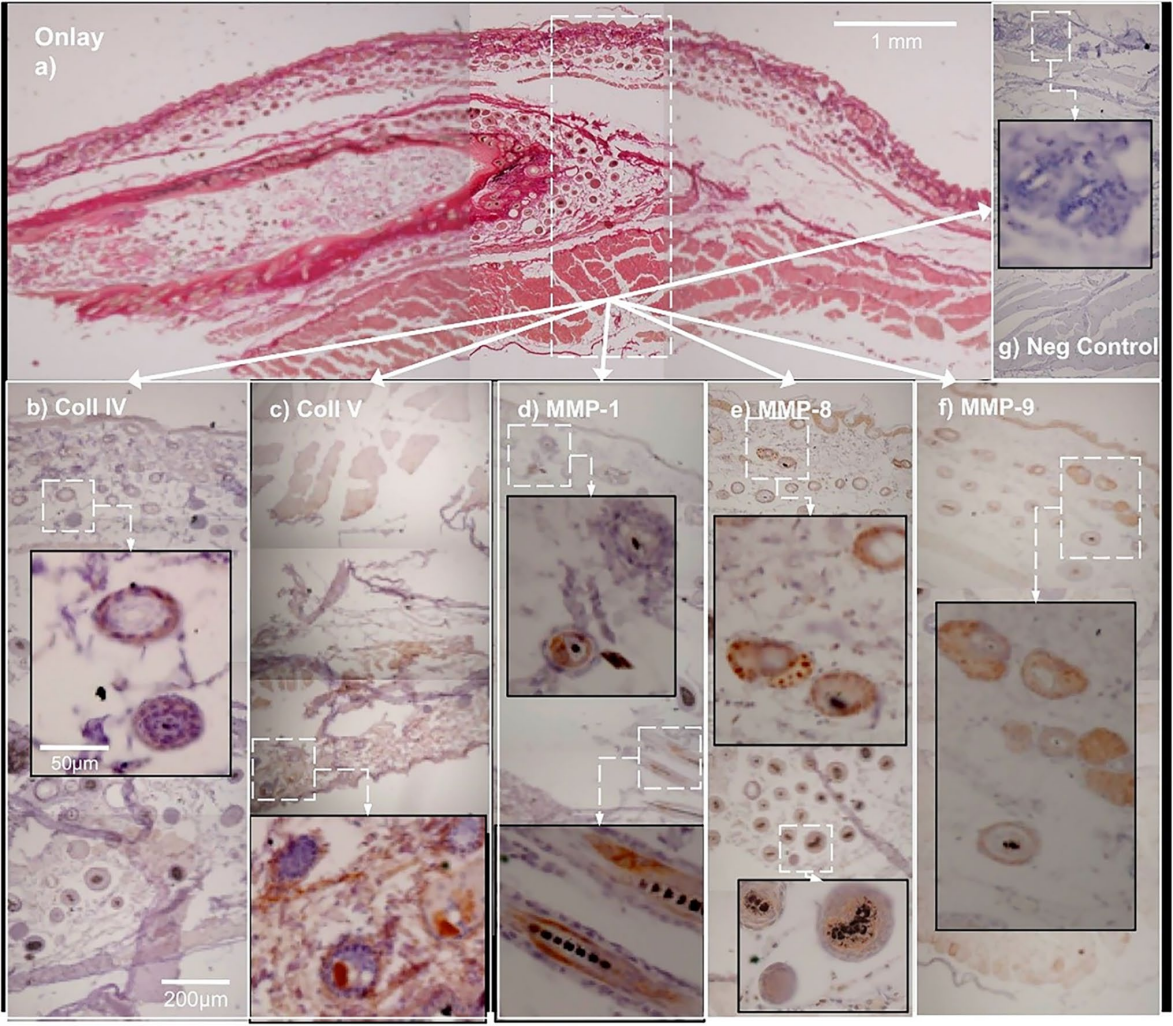
Detail of transition zones around onlay grafts with IHC staining for collagen and MMP (**a**–**f**) compared to negative control (**g**). Collagen type IV (**b**) and type V (**c**) do not show co-localization with MMP-1 (d), MMP-8 (**e**) or MMP-9 (**f**). The size bar equals 1 mm (**a**), 200 µm (**b**–**g**) and 50 µm in inserts of **b**-**g**

**Fig. 5:**
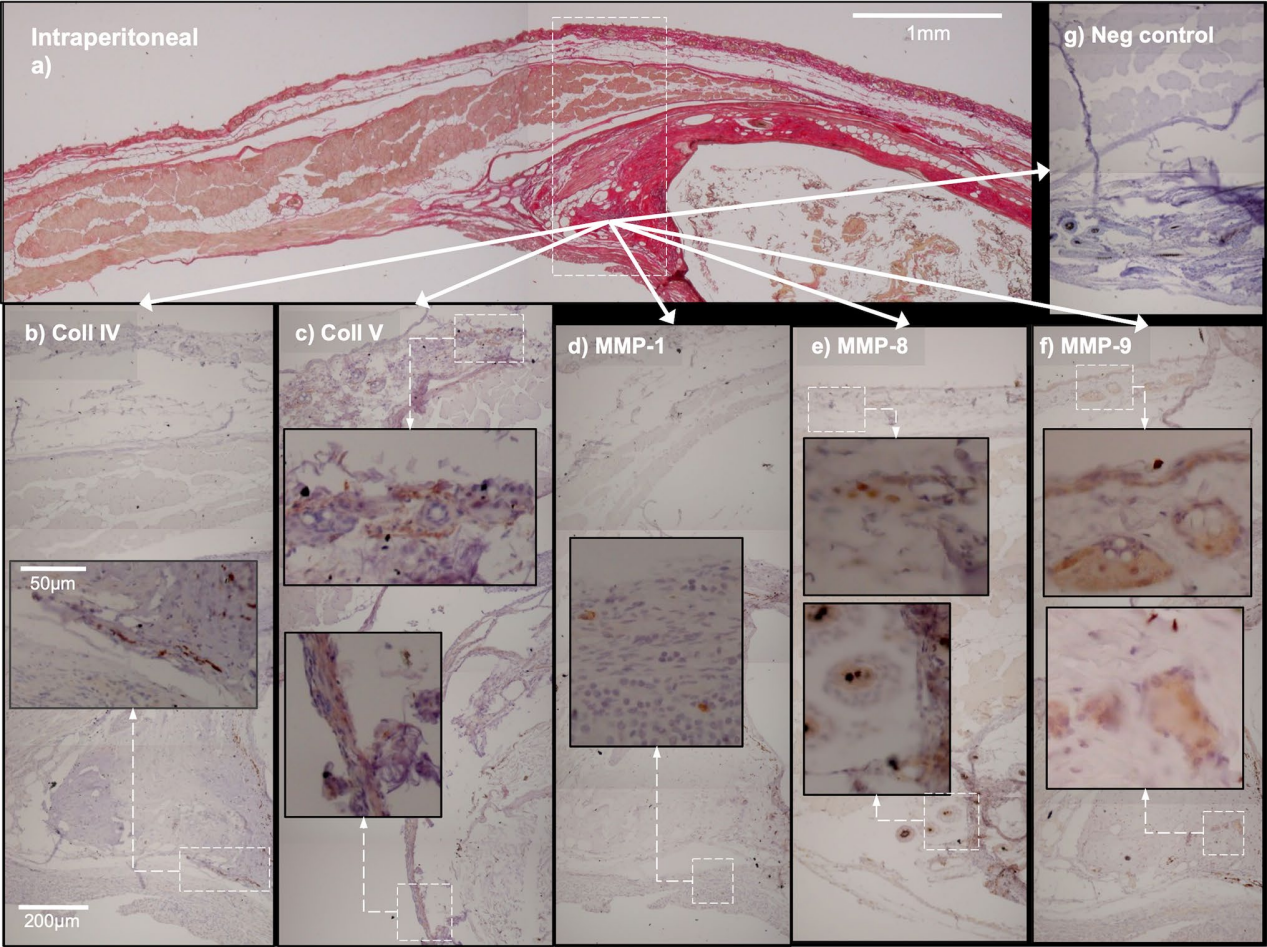
Detail of transition zones around intraperitoneal grafts with IHC staining for collagen and MMP (**a**–**f**) compared to negative control (**g**). Collagen type IV (**b**) and type V (**c**) do not show co-localization with MMP-1 (**d**), MMP-8 (**e**) or MMP-9 (**f**). The size bar equals 1 mm (**a**), 200 µm (**b**–**g**) and 50 µm in inserts of (**b**–**g**)

Antibody staining for MMP-1, MMP-8 and MMP-9, collagen type IV and collagen type V, viewed at × 40, showed mainly hair follicles and their glands, with no obvious co-localization (Figs. 4, 5). Since IHC-stained sebaceous glands and hair follicles were not observed in areas of interest within FTSGs, no semi-quantification was carried out.

## Discussion

The main hypothesis was upheld. FTSG applied in the intraperitoneal and onlay positions showed similar structural appearances in this cross-sectional descriptive study, with an abundance of thick collagen bundles in the dermis compared to dermis in internal controls. Surprisingly IHC staining of collagens and MMP was scarce, with no obvious co-localization. Since no systematic differences were seen between the two FTSG implantation sites, it follows that no difference in biomechanical behavior can be anticipated between FTSGs in the intraperitoneal and onlay positions.

The thick collagen type I bundles revealed by Picrosirius staining, were more conspicuous in the dermis of FTSGs than in the dermis of their internal control. A decrease in the collagen type I/III ratio has been described in several studies on different types of hernia [24–26], and may be a key factor in hernia development. Increase in thin green-birefringent collagen bundles corresponding to collagen type III was not seen in the FTSGs, suggesting tissue strength of the FTSG [14], which would be beneficial in reinforcing abdominal wall integrity.

The second hypothesis was upheld. Metamorphosis to thicker collagen type I bundles in the dermis with partially degraded skin appendages as seen in the FTSGs, is in line with previous observations in the onlay position [6, 27, 28], where continual degradation of hair follicles and sebaceous glands has been observed over several months [28]. This may translate into increased tissue strength, but no biomechanical studies could be performed in this study due to the limited number of animals. However, very promising tests on tissue strength have been performed on FTSG samples, showing tensile strength superior to both synthetic and biologic implant materials [8], but this has yet to be confirmed in long-term experiments.

The third hypothesis was rejected since no obvious co-localization of MMP with their substrate collagens type I, III, IV or V was seen (Figs. 4, 5). This is surprising, since it was expected that MMP together with their substrates would be seen together in transitional zones where wound healing/regeneration and matrix remodeling was expected to take place. Previous research has suggested that MMP remodulation takes place in the early inflammatory phase of wound healing while tissue maturation and remodeling occur several months to years after injury [29]. But low expression of MMP-1 may imply a low inflammatory response and stable connective tissue turnover, since increased MMP-1 levels triggered by TNF-α in vitro have been associated with instability in carotid plaque and subsequent rupture due to matrix remodulation [30]. However, MMP-1 have been associated with both proinflammatory and anti-inflammatory actions through processing of IL-1β [15], impeding far-reaching conclusions. Interpretation of collagen types IV and V staining was hampered by poor tissue morphology since horseradish peroxidase pockets generate false positive staining. Some expression was observed in transitional zones, but no overt overexpression of collagen type V (Figs. 4c, 5c), which has been associated with inflammation, granulation tissue and several forms of fibrosis, was seen [31]. On the other hand, absence of staining may imply collagen deficiency which in Ehler-Danlos syndrome is associated to decreased tissue strength [31]. In a cross-sectional study, each slide represents a snapshot of a continuous process, visualizing the ongoing biologic process at one point in time only. There is thus a risk that some steps in tissue remodeling and repair have already taken place or have not yet begun. Another possible explanation is that remodeling is in progress but via mediators other than those monitored in the present study.

Due to the natural posture of mice i.e. four-legged and forward bent, the tension on a graft during ingrowth is uncertain. During surgery, the mice were placed on their back with the abdomen stretched out. Application of a FTSG under tension has been considered crucial to avoid dermoid cyst formation and to achieve tissue metamorphosis [27]. It has also been considered important to closely adapt tissues to avoid seroma formation. This was not possible in the present experimental model due to the restricted surgical field available and may explain the observation of dermoid cysts. In a clinical study on FTSG use in hernia repair, biopsies taken later from onlay transplants did not show dermoid cysts [6].

IHC staining has several sources of error including non-specificity of antibodies [32]. In this case, however, it seems unlikely that the absence of IHC expression in grafts represents a false negative result since negative control samples did not bind antibodies. Furthermore, internal controls showed increased staining in a gradient dependent manner in vascular structures, hair follicles and their glands.

Picrosirius Red (Sirius Red F3BA) has been shown to stain connective tissue in a birefringent manner [33], with an orange/red color associated with thick collagen type I fibers and green birefringence with thin collagen type III fibers [21]. One paper claimed that discrimination between collagen type I and III in polarized light could be an effect of inherent difference in birefringence of the tissue in combination with positioning of the polarization filter [34]. This effect, however, was not seen in the present study. Even if intensities of the orange/red and green areas varied to some extent, the color distribution was constant with no change from green to orange/red, indicating no difference in birefringence due to positioning of the polarizing filter. Furthermore, Junqueira et al. have reported that green birefringence is only seen in tissues containing collagen type III, which supports the specificity of this staining [21].

The relatively disappointing long-term results of acellular biologic mesh may possibly be due to animal epitopes remaining in collagen networks causing a late foreign body response towards the structure i.e. a delayed type IV sensitivity reaction [35]. Since autologous FTSGs have identical MHC class 2 proteins, they should not trigger a foreign body reaction due to MHC class 2 mismatch. However, an exaggerated sterile inflammation may cause a similar reaction [36], but this was not seen in the present material.

Despite failure to detect target epitopes, the present observation that collagen distribution is similar in FTSG in intraperitoneal and onlay positions is important as it implies a similar course of events takes place in both positions. FTSG in the onlay position in hernia repair has already been shown to be successful up to one year in humans, with less postoperative abdominal pain compared to synthetic mesh, even if no differences in recurrence was seen [6, 9].

One of the main strengths of this study is that the area of each sample examined was large, thereby limiting the risk of omitting important regional processes. Although there are differences between human and mice tissue biology, ECM components and collagen composition are similar between the two species and the mouse model is widely used for research on skin disease [37]. Another strength is that the mouse intervention model used resembles human clinical practice as far as possible.

The importance of this study is the systematic characterization of FTSG morphology in the intraperitoneal position compared to that in the onlay position; a precautionary measure prior to use of FTSG in the intraperitoneal position in humans. This confirms the findings of a basic clinical animal study showing no FTSG-associated adverse events in a population of mice [10], thereby adding to the evidence that use of FTSG is relatively safe, albeit the small number of mice in this study. These encouraging results endorse further confirmation in a human clinical setting. The study also provides necessary information for calculating group size in future studies on safety and long-term effects. Regular mesh parameters presumably apply to this graft concept in terms of overlap and mechanical stability albeit all these parameters need dedicated studies. Furthermore, the maximum FTSG size need further investigation, although the FTSG covered about 25% of the mice abdominal wall area, straight extrapolation to humans Viability of larger FTSG may be preserved through blood vessels entering the autologous tissue, which hypothetically could increase the quality of FTSG integration. A low inflammatory response, supposedly due to autologous tissue, may be one reason why lower postoperative pain was observed in a previous study [6, 9].

Together with previous results from implantation in the onlay position [6, 9], verified 100% graft survival [10], low inflammatory response and very few adhesions [10], the present results encourage further research on FTSG application in hernia repair. Controlled clinical trials on FTSG applied in the intraperitoneal position in laparoscopic hernia repair and complicated parastomal hernia repair are urgently required. Increase in our knowledge of biologic mechanisms involved in tissue remodeling and repair after application of FTSG in abdominal wall reconstruction increases our ability to use autologous grafts in several positions with wider surgical indications, providing new options in the hernia surgeon’s toolbox.

## Data Availability

All data generated or analysed during this study are included in this article. Further enquiries can be directed to the corresponding author.
