# Correction to: Similar collagen distribution in full-thickness skin grafts in intraperitoneal and onlay positions, an experimental mice-study

**DOI:** 10.1007/s10029-022-02728-1

**Published:** 2022-12-03

**Authors:** A. Winsnes, M.-L. Ivarsson, P. Falk, U. Gunnarsson, K. Strigård

**Affiliations:** 1 Department of Surgical and Perioperative Sciences, Surgery, Umeå University, Daniel Naezéns väg, 901 87 Umeå, Sweden; 2 Department of Surgery, University of Gothenburg, Gothenburg, Sweden

**Correction to: Hernia (2022) 26:1695–1705** 10.1007/s10029-022-02664-0

The funding note which currently reads:

“This work was supported by federal grants: The Swedish Research Council 2017-00824”

But it should read as The Swedish Research Council 2021-00972.

The authors apologies for this mistake.

The original article has been corrected.

